# Workmen's Compensation Cases

**Published:** 1910-11-05

**Authors:** 


### Medico-Legal Points.

### WORKMEN'S COMPENSATION CASES.

In a case at Wigan, on May 13, Judge Bradbury had something to say as to the proper course to be adopted in case two doctors differed. The applicant, on February 18 last year, was struck by a fall of stone and received serious injuries to his back and left side. He was paid compensation until June 24 last year, and recommenced work of a light character. In November he ceased work in consequence of the injury of February. The defence, in that case, was that any incapacity which the applicant was now suffering was not the result of the accident. He had two medical men present who were prepared to give a very strong opinion, amounting, in their view, to a certainty that the applicant was suffering as a result of the accident and was incapacitated from work. The respondent also had two witnesses, medical men, who were going to express a contrary view in support of the defence. His Honour said the question was whether the heart disease or the bronchitis was attributable to the accident. In his opinion, the position was this: When they called a medical man his evidence was final, because his opinion was that of a skilled medical man, and it was evidence he was asked to rely upon. When that one man's evidence was given he could accept it, but, supposing diametrically opposite opinion was given, then he had two opinions, upon each of which he could rely. If they were diametrically opposed to each other, he ceased to rely upon the evidence. In that case two doctors were going to say it was spinal disease, and two others would say it was not. He suggested some form of reference should be drawn up and submitted to the Medical Referee.

### An Eye Case.

In a case at Bristol it appeared that in September 1909 a man who worked for a colliery company was hewing a knot off a piece of timber, when the knot flew and struck him with force on the right eye. On the Monday following Dr. Nevill removed a splinter from the eye. From the date of the accident compensation at the rate of 9s. 7d. per week had been paid up to November 4. On that date the man applied for work, but his application was not complied with, and the applicant had not been found any work by the company since. His Honour said that really the only question was whether the applicant was incapacitated through the accident as from November 4. The applicant said he was not aware that he squinted before the accident. He was willing to do work if a light job was found for him, but when he applied the manager said he could not keep him there on compensation for the rest of his days. He could take a job on the bank if it did not involve running about. His knees gave him trouble. He had splendid sight before the accident.

Dr. Mitchell said his examination of the eye revealed a slight open wound on the surface; there was an astigmatism, which might have been caused by the accident or might be congenital. His opinion was that the squint was not due to the accident, but was of long standing. A doctor, called for the respondents, said that the applicant's eye had recovered from the accident. The left eye had never been much good to the applicant, and he could see now, in his opinion, just as well as before the accident. The squint, he believed, was of long standing, and not due to the accident. His Honour said he could not hold that applicant had proved beyond doubt that the accident was the cause of his incapacity for work. The application was dismissed with costs.

